# Conditional deletion of neurogenin-3 using *Nkx2.1iCre* results in a mouse model for the central control of feeding, activity and obesity

**DOI:** 10.1242/dmm.011916

**Published:** 2013-05-02

**Authors:** Neal Anthwal, Michelle Pelling, Suzanne Claxton, Georg Mellitzer, Caitlin Collin, Nicoletta Kessaris, William D. Richardson, Gérard Gradwohl, Siew-Lan Ang

**Affiliations:** 1 Division of Developmental Neurobiology, MRC-National Institute for Medical Research, London, NW7 1AA, UK; 2 IGBMC, INSERM U964, CNRS UMR 7104, Université de Strasbourg, 67404 Illkirch, France; 3 Wolfson Institute for Biomedical Research, Research Department of Cell and Developmental Biology, University College London, London, WC1E 6BT, UK

## Abstract

The ventral hypothalamus acts to integrate visceral and systemic information to control energy balance. The basic helix-loop-helix transcription factor neurogenin-3 (Ngn3) is required for pancreatic β-cell development and has been implicated in neuronal development in the hypothalamus. Here, we demonstrate that early embryonic hypothalamic inactivation of *Ngn3* (also known as *Neurog3*) in mice results in rapid post-weaning obesity that is associated with hyperphagia and reduced energy expenditure. This obesity is caused by loss of expression of *Pomc* in *Pomc*- and *Cart*-expressing (*Pomc*/*Cart*) neurons in the arcuate nucleus, indicating an incomplete specification of anorexigenic first order neurons. Furthermore, following the onset of obesity, both the arcuate and ventromedial hypothalamic nuclei become insensitive to peripheral leptin treatment. This conditional mouse mutant therefore represents a novel model system for obesity that is associated with hyperphagia and underactivity, and sheds new light upon the roles of Ngn3 in the specification of hypothalamic neurons controlling energy balance.

## INTRODUCTION

A number of interconnected hypothalamic nuclei are involved in the central control of energy balance. These include the arcuate nucleus (ARC), ventromedial hypothalamic nucleus (VMH), lateral hypothalamic area (LH), dorsomedial hypothalamic nucleus (DMH) and periventricular hypothalamic nucleus (PVN). The ARC and VMH are exposed to the circulation owing to active transport of metabolic signals such as leptin, glucose and insulin across the blood-brain barrier. These signals are then detected in the ARC and VMH due to the presence of transmembrane receptors. Within the ARC, there exists two related but exclusive neuronal populations regulating energy balance: the neurons expressing proopiomelanocortin (*Pomc*) and cocaine- and amphetamine-regulated transcript (*Cart*), and those expressing neuropeptide Y (*Npy*) and agouti related peptide (*Agrp*). *Pomc*/*Cart* neurons are located within the lateral domain of the ARC and act to inhibit feeding activity, whereas *Npy/Agrp* neurons are expressed more medially, and act to promote feeding. *Pomc/Cart* neurons are a heterogeneous population, with distinct subpopulations capable of responding to different signals, such as leptin via the leptin receptor (LepR), glucose via glucose-sensitive potassium (K^+^) channels such as Sur1 and Kir6.2, and insulin (Cowley et al., 2001; Ibrahim et al., 2003; Parton et al., 2007; Williams et al., 2010). Furthermore, during development, *Pomc* expression is observed in postmitotic neuronal precursors between embryonic day 10.5 (E10.5) and E14.5, only 50% of which give rise to mature *Pomc/Cart* neurons, the others maturing into other ventral hypothalamic subtypes, including *Npy/Agrp* arcuate neurons (Padilla et al., 2010).

The other hypothalamic nucleus involved in the primary detection and integration of systemic information into behavioral response to control energy balance is the VMH. This nucleus is defined by the expression of steroidogenic factor 1 (SF1), a nuclear receptor transcription factor encoded by *Nr5a1* that is known for its function in gonadogenesis and adrenal steroidogenesis (Parker and Schimmer, 1997). SF1 expression is confined to the VMH in the mature hypothalamus; however, *Pomc*-expressing postmitotic precursors also transiently express SF1 during embryonic development (Pelling et al., 2011). As in the ARC, VMH neurons have been shown to detect the circulating hormones leptin, glucose and insulin (Dhillon et al., 2006). The deletion of LepRs in SF1-expressing cells results in adult-onset obesity with an associated reduction in energy expenditure prior to the onset of obesity (Bingham et al., 2008).

Although the importance of ARC and VMH neurons in the control of feeding and activity is well established, little is known about their development. This is potentially important, because there are variations in the incidence of obesity in populations in the developed world that experience similar diet and lifestyles, suggesting the existence of still-unknown genetic and/or developmental influences on the tendency to develop obesity (Ramachandrappa and Farooqi, 2011). Recent studies in rodents have demonstrated that the maternal diet during gestation and lactation has an influence on the later expression levels of the arcuate-derived neuropeptides involved in feeding in the offspring (Chen et al., 2008; Chen et al., 2009). The development and genetic basis of human obesity is still poorly understood (Ramachandrappa and Farooqi, 2011). Further knowledge might help in the prevention and treatment of obesity and its associated metabolic disorders, such as type 2 diabetes. In addition to understanding the genetic basis of obesity, mechanistic insights into the development of the hypothalamus will illuminate how the developmental environment is able to influence the activity of the adult hypothalamus.

**TRANSLATIONAL IMPACT**

**Clinical issue**

The prevalence of obesity in Western societies is reaching epidemic proportions. Although diet and lifestyle are the main contributors to the rise in obesity, variation within populations indicates that the genetic and developmental environment can influence an individual’s susceptibility to obesity. The hypothalamus is an area in the brain that is involved in the regulation of energy balance. Despite advances in our understanding of its role, relatively little is known about hypothalamic development. The transcription factor neurogenin-3 (Ngn3) has been implicated in neuronal development in the hypothalamus. Enhancing our understanding of the mechanisms underlying hypothalamus development via studies in mice will shed light on the role of embryonic development in human obesity.

**Results**

In this work, the authors present a novel mouse model of obesity. The model was generated by hypothalamic deletion of *Ngn3* (also known as *Neurog3*), which results in rapid post-weaning obesity, concomitant with increased food intake (hyperphagia) and reduced energy expenditure. Because Ngn3 is an important factor in insulin-producing pancreatic β-cell development, global mutants for *Ngn3* die shortly after birth owing to severe diabetes. However, in the model presented here, the genetic insult is restricted to the brain, so these mice are born with normal insulin production. Following the onset of obesity, mutant mice develop insulin resistance, increased glucose tolerance, and changes in blood biochemistry such as increased leptin, adrenocorticotropic hormone and insulin levels. The authors identified the primary cause of obesity as loss of hypothalamic *Pomc* expression, which is known to induce hyperphagia, whereas other energy-balance-related hypothalamic peptides were unaffected. The authors also identified changes that could mediate the reduction in energy expenditure in obese mice, notably insensitivity of the ventromedial hypothalamus to leptin.

**Implications and future directions**

These data indicate that Ngn3 is likely to have roles in specifying hypothalamic control of both sides of the energy balance equation. Further analysis will provide mechanistic insights into the role of Ngn3 in the specification of the hypothalamus. Ngn3 and its currently undefined targets represent promising candidates for the prevention of developmentally determined obesity. The current study also provides a novel obesity model in which extreme levels of obesity are achieved with no impact on peripheral tissues. As such it offers an improved system compared with classical models such as the leptin-signaling-deficient *ob/ob* and *db/db* mice. Similarly, the model facilitates the study of *Pomc* disruption specifically in the hypothalamus, circumventing the global effects associated with the deletion of this gene in the pituitary gland.

The basic helix-loop-helix (bHLH) transcription factor neurogenin-3 (Ngn3) is an important player in the development and specification of the endocrine control of energy balance in mice. Ngn3 acts with its partner, Pdx1, in the specification of insulin-producing β-cells in the pancreas (Gradwohl et al., 2000; Gasa et al., 2004; Schonhoff et al., 2004; Smith et al., 2004; Johansson et al., 2007). It also acts to specify the endocrine cells of the gastric and intestinal epithelium (Jenny et al., 2002). An intestine-specific deletion of *Ngn3* (also known as *Neurog3*) in mice results in a failure to produce a number of gut enzymes, including those involved in lipid metabolism, resulting in a lean phenotype and death in early adulthood from malnutrition (Mellitzer et al., 2010). Furthermore, there are a number of reported point mutations of *NGN3* in humans that result in severe neonatal malabsorptive diarrhea and infant diabetes (Wang et al., 2006; Pinney et al., 2011).

Lineage studies indicate that *Ngn3*-expressing cells contribute to ventral hypothalamic areas such as the ARC and VMH (Pelling et al., 2011), but not to the more dorsal DMH or PVN (data not shown). We have previously described a role for Ngn3 and another bHLH transcription factor, Ascl1 (also known as Mash1), in the development of the mouse hypothalamus (McNay et al., 2006; Pelling et al., 2011). Interestingly, Ngn3 regulates the development of ARC nucleus neurons. Loss of *Ngn3* in mice results in an increase in the number of *Npy*^+^ neurons, accompanied by a severe loss of *Pomc*^+^ neurons. However, *Ngn3*-null mutant mice die shortly after birth owing to the role of Ngn3 in pancreatic β-cell specification, and the consequence of *Ngn3* deletion in the developing hypothalamus upon the behavior of adult mice could not be assessed. In the present study, we use a conditional knockout system to specifically delete *Ngn3* from the ventral forebrain, resulting in a depletion of *Pomc* expression in ARC neurons, a decrease in leptin sensitivity in the ARC and VMH nuclei, and an associated obesity due to hyperphagia and reduced energy expenditure. Using this new conditional *Ngn3* mouse model, we also demonstrated that Ngn3 is required for the expression of *Pomc* and nescient helix loop helix 2 (*Nhlh2*), but not for the expression of *Cart* in most *Pomc/Cart* neurons.

## RESULTS

### Generation of *Ngn3* conditional-knockout mice

**Fig. 1.**
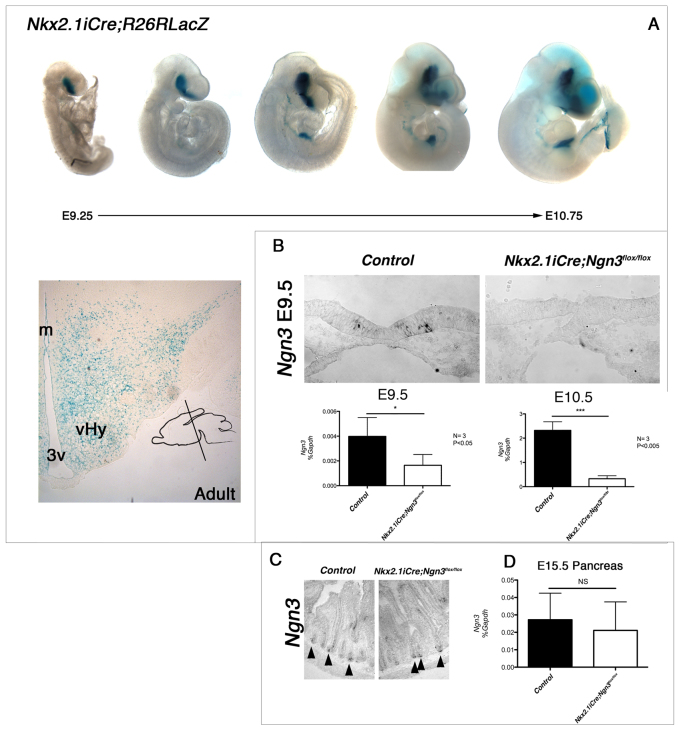
***Nkx2.1iCre* drives *Ngn3* deletion in the ventral forebrain.** (A) Expression of *Nkx2.1iCre* in the developing mouse embryo. *R26RLacZ* reporter females were crossed with *Nkx2.1iCre* males, and resulting embryos stained with X-Gal to reveal *lacZ* reporter expression. β-gal-positive cells are observed in the ventral forebrain as early as E9.25 and throughout the adult hypothalamus. β-gal-positive cells are also observed in endodermal-derived tissues from around E10.0. Insert represents angle of section throughout study. m, midline; 3v, third ventricle; vHy, ventral hypothalamus. (B) *Ngn3* was conditionally deleted in *Nkx2.1*-expressing cells by crossing *Nkx2.1iCre* males with *Ngn3^flox/flox^* females, resulting in *Ngn3* conditional mutant mice (*Nkx2.1iCre/+;Ngn3^flox/flox^*). Following deletion, *Ngn3* expression was not observable by *in situ* hybridization at E9.5. However, qRT-PCR reveals low levels of *Ngn3* in the E9.5 ventral forebrain, whereas only residual levels are seen at E10.5. Student’s *t*-test: *P<0.05 and ***P<0.005. (C,D) Despite iCre-positive cells being observed in the developing endoderm, no change was seen in the expression of *Ngn3* by *in situ* hybridization in the adult intestinal crypt cells (C), nor by qRT-PCR in the developing pancreas (D). Arrowheads in C indicate Ngn3-positive crypt cells. NS, not significant.

To study the consequence of the loss of Ngn3 in the developing hypothalamus on energy balance in adult animals, we used *iCre* driven by the *Nkx2.1* promoter to delete *Ngn3* in *Nkx2.1iCre/+;Ngn3^flox/flox^* mice. The domain of *Ngn3* deletion was confirmed by crossing *Nkx2.1iCre/+* mice with *R26R* homozygous mice, which contained floxed *lacZ* alleles in the Rosa26 locus (Soriano, 1999) in order to trace the location of iCre recombinase activity. Embryos were dissected and stained with X-Gal solution to reveal the presence of the *lacZ* reporter. In the developing CNS, strong β-galactosidase (β-gal) expression was seen in the ventral forebrain as early as E9.25, similar to the onset of expression of Nkx2.1 (Kimura et al., 1996; Sussel et al., 1999) and also of Ngn3 (Fig. 1A). In the mature nervous system, β-gal expression was observed throughout the hypothalamus, including the ARC, VMH and lateral hypothalamic area (Fig. 1A). β-gal expression was also seen in the developing gut endoderm and lung anlage. In order to confirm that *Ngn3* deletion was restricted to the forebrain, quantitative reverse-transcriptase PCR (qRT-PCR) for *Ngn3* was carried out on E15.5 pancreatic tissue (Fig. 1C), and *in situ* hybridization for *Ngn3* was carried out on the adult small intestine. *Ngn3* expression in these two endodermal tissues of *Nkx2.1iCre/+;Ngn3^flox/flox^* mice was the same as seen in these tissues in control littermates (*Ngn3^flox/flox^*) (Fig. 1D). Furthermore, adult *Nkx2.1iCre/+;Ngn3^flox/flox^* mice did not exhibit the gut and pancreas abnormalities associated with a null mutation of *Ngn3*. In order to confirm brain deletion of Ngn3, we analyzed *Ngn3* expression by *in situ* hybridization of E9.5 control and *Nkx2.1iCre/+;Ngn3^flox/flox^* embryos. Whereas control embryos showed *Ngn3* expression in ventral hypothalamic progenitors, no signal was detected in the same region of *Nkx2.1iCre/+;Ngn3^flox/flox^* embryos. Further confirmation of deletion was sought by qRT-PCR analysis of *Ngn3* expression in dissected ventral forebrain tissue at E9.5 and E10.5. qRT-PCR revealed a residual *Ngn3* expression in the ventral forebrain of *Nkx2.1iCre/+;Ngn3^flox/flox^* at E9.5 (Fig. 1B), whereas, by E10.5, expression levels were reduced to background level.

### Hypothalamic *Ngn3* deletion results in early-onset obesity accompanied by hyperphagia and reduced adult activity

**Fig. 2.**
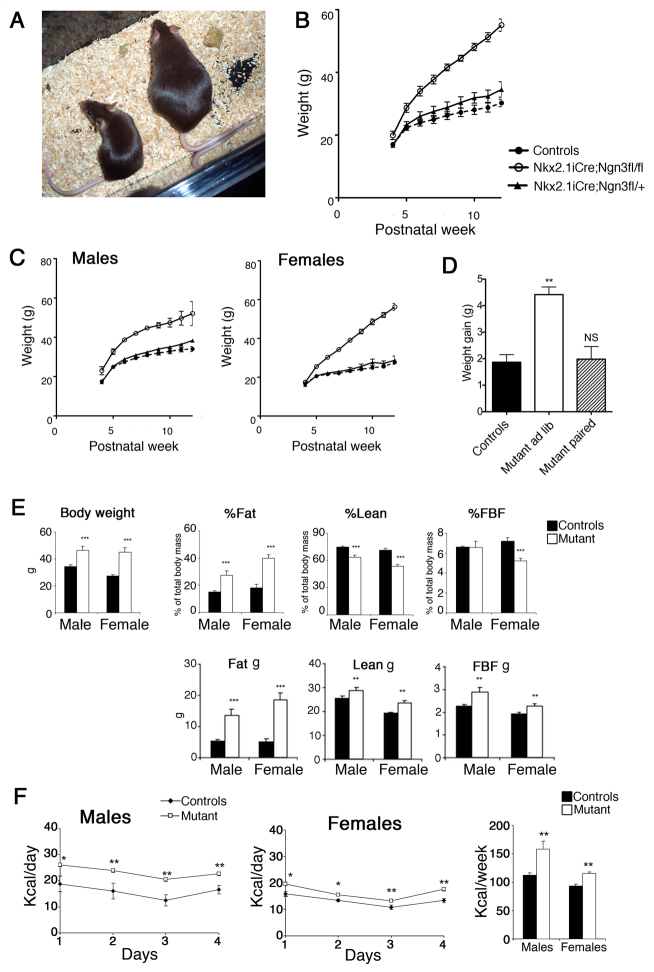
***Nkx2.1iCre/+;Ngn3^flox/flox^* mice become viscerally obese and are hyperphagic.** (A) Examples of control (left) and conditional mutant (right) 8-week-old female littermates. (B,C) Mutant *Nkx2.1iCre/+;Ngn3^flox/flox^* mice become significantly heavier than control and *Nkx2.1iCre/+;Ngn3^flox/+^* littermates from postnatal week 5 (around 1 week after weaning), and continue to gain mass well into mature adult stages. The rate of weight gain is more rapid in females than males (C). (D) *Ad libitum*-fed *Nkx2.1iCre/+;Ngn3^flox/flox^* mice gain more weight per week between 4 and 6 weeks of age than do *Nkx2.1iCre/+;Ngn3^flox/flox^* mutants pair-fed to control littermates. (E) Body weight, fat mass, lean mass and free body fluids (FBF) presented as percentage body weight (%) and as total mass (g) in 14-week-old control and mutant male and female mice. Body weight and proportion of body mass made up of fat tissue is significantly increased in mutant animals, whereas the percentage lean mass is reduced. The percentage free body fluid is significantly reduced in female mutant mice compared with controls, but not in males. (F) Both male and female *Nkx2.1iCre/+;Ngn3^flox/flox^* mice show a significant increase in daily and cumulative weekly food intake. Student’s *t*-test: *P<0.05, **P<0.01, ***P<0.005; NS, not significant.

We have previously shown a severe loss of *Pomc*^+^ neuronal number followed by increased numbers of *Npy*^+^ neurons in the ARC of *Ngn3^−/−^* embryos during development (Pelling et al., 2011). Owing to the importance of these circuits in energy balance, we therefore investigated the body mass, food intake and energy expenditure of *Nkx2.1iCre/+;Ngn3^flox/flox^* mice. In contrast to the conventional knockout of *Ngn3*, *Nkx2.1iCre/+;Ngn3^flox/flox^* mice survived to adulthood, with no apparent increase in juvenile mortality. At weaning, control and mutant mice were morphologically similar; however, shortly after weaning mutant mice rapidly became obese, with homozygous mutant mice being significantly heavier than control and *Nkx2.1iCre/+;Ngn3^flox/+^* littermates by postnatal week 5 (Fig. 2A,B). This weight gain continued well into sexual maturity, with both male and female mice being fertile and capable of producing viable offspring. The difference in mass was more pronounced in females than in males, and the rate of increase in body weight also continued at a greater rate in females (Fig. 2C). Mutant mice (*n*=3) were pair-fed against control littermates between 4 and 6 weeks of age. The mean weekly increase in body weight was significantly high in *ad libitum*-fed mutant animals when compared with control littermates, whereas pair-fed mutant animals showed no difference to control littermates (Fig. 2D). A cohort of 18 control (9 male and 9 female) and 18 mutant (9 male and 9 female) mice were analyzed for differences in metabolism and physiology (*n*=9 for each sex and genotype). Body weights were recorded once a week from the age of 12 to 22 weeks. In this group, both male and female mutant mice displayed significantly higher body weights compared with controls, and the difference was more pronounced in females than in males. In order to ascertain body composition, quantitative nuclear magnetic resonance (qNMR) imaging was carried out at 14 weeks of age (Fig. 2E). The proportion of body weight made up of fat mass was increased, whereas the proportion of lean mass decreased. The total mass of lean tissue increased slightly but significantly. Taken together, these data demonstrate that elevated body mass is mainly due to an increase in adipose tissue (Fig. 2E). The proportion of body weight made up of free fluid mass was significantly decreased in mutant females compared with controls, whereas no such difference was observed in males (Fig. 2E). At 12 weeks of age, daily food intake was measured. Following 7 days of experimental habituation, the difference in daily food intake was significantly higher in both male and female mutant mice compared with controls (Fig. 2F). The cumulative weekly food intake was significantly higher in mutant mice (Fig. 2F) and, although significance was achieved in both male and female mice, the increase was larger in males.

**Fig. 3.**
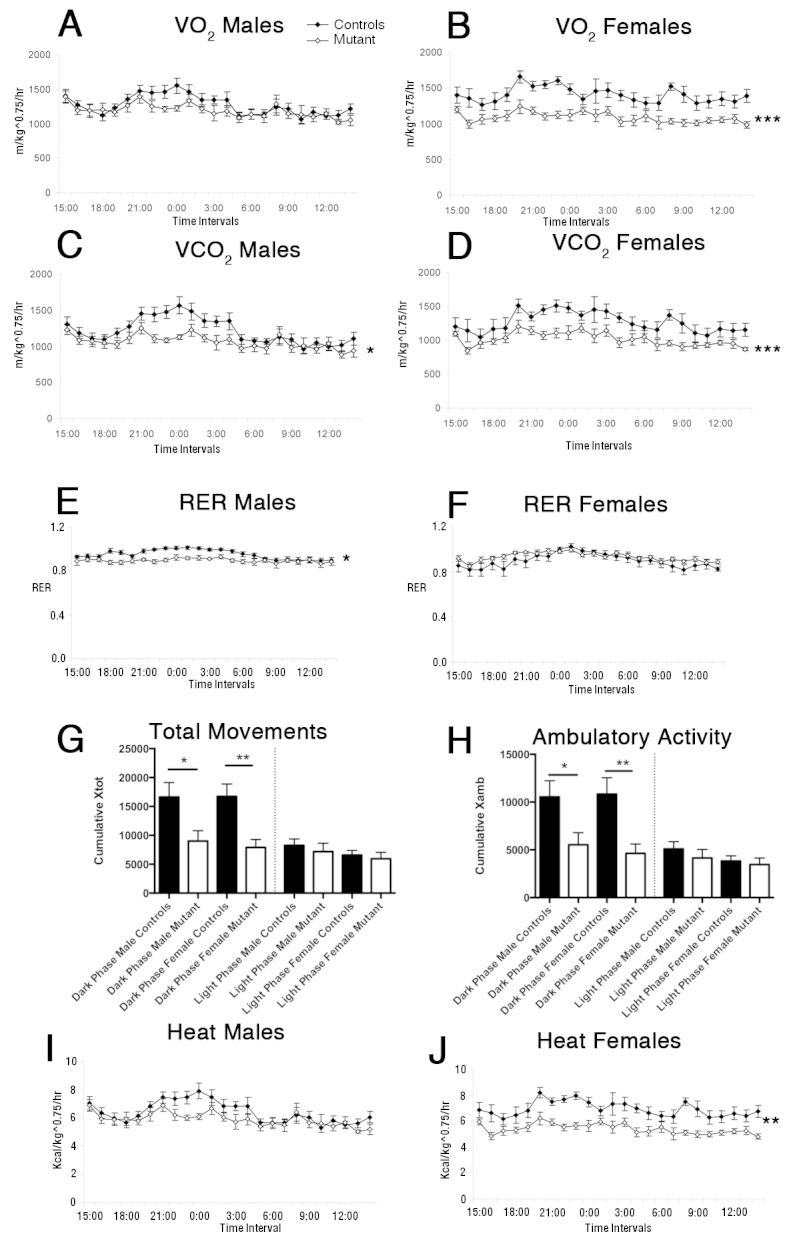
***Nkx2.1iCre/+;Ngn3^flox/flox^* mutant mice exhibit decreased energy expenditure at 17 weeks of age compared with controls.** (A,B) Oxygen consumption in male and female mice. There is a trend for decreased oxygen consumption in mutant male mice compared with control mice particularly in the night-time dark phase (A), whereas, in female mutant mice, oxygen consumption if significantly reduced compared with controls (B). (C,D) CO_2_ production is significantly reduced in both male (C) and female (D) mutant mice compared with control littermates. (E,F) Respiratory exchange ration (RER) is significantly lower in male mutant mice compared with controls (E); no difference is observed in females (F). (G,H) Movement, measured by total movement (G) and ambulatory activity (H), is significantly reduced in mutant mice during the dark phase but not during the light phase. (I,J) There is a reduction in heat production in mutant mice. Male mice show a trend of reduced heat production compared with controls, particularly in the dark phase (I), whereas heat production is consistently reduced in female mutant mice (J). *P<0.05, **P<0.01, ***P<0.005.

Energy expenditure was evaluated by indirect calorimetry and measurement of ambulatory activity in the same cohort at 17 weeks (*n*=9 for each sex and genotype). Both male and female mutant mice displayed a reduction in oxygen consumption and carbon dioxide production (Fig. 3A–D). In males, oxygen consumption (VO_2_) tended to be lower, particularly during the night (Fig. 3A) and carbon dioxide production (VCO_2_) was significantly lower (Fig. 3C). In addition, male mutant mice showed a lower respiratory exchange ratio (RER) than controls (Fig. 3E). Female mutant mice displayed significantly lower VO_2_ and VCO_2_ levels than the controls both during the day and night period (Fig. 3B,D), but their RERs were similar to the controls (Fig. 3F), in part owing to variability within the mutant group. Ambulatory activity and total movements were lower in both male and female mutant mice compared with controls during the dark phase, but not during the light phase (Fig. 3G,H), as was heat production (Fig. 3I,J).

Taken together, these data demonstrate that loss of *Ngn3* during development leads to rapid post-weaning obesity associated with hyperphagia and reduced energy expenditure.

### Hypothalamic deletion of *Ngn3* results in insulin insensitivity and altered blood biochemistry

**Fig. 4.**
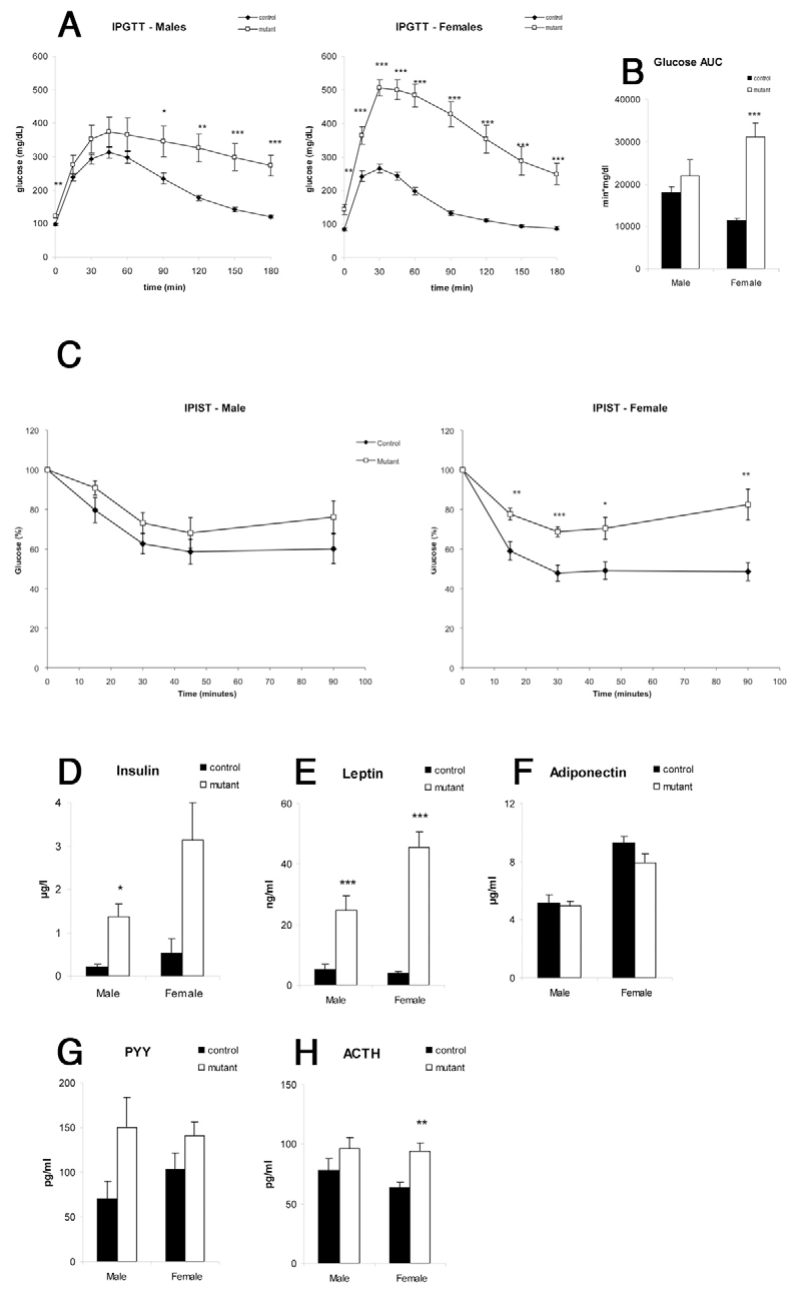
***Nkx2.1iCre/+;Ngn3^flox/flox^* mice display reduced glucose tolerance and insulin sensitivity, and show changes in blood hormone levels compared with controls.** (A,B) Intraperitoneal glucose tolerance test (IPGTT). White squares, mutants; black squares, controls. Female mutant mice showed significantly higher glucose levels at all time points of the test, whereas, in male mutants, levels were not significantly higher immediately following injection; however, the glucose decrease following glycemic peak is slower (A). Female mutant mice showed significantly higher area under glucose curve (AUC) than controls (B). (C) Insulin sensitivity test (IPIST). Female mutant mice are significantly insulin resistant, whereas male mice do not reach significance. (D–H) Blood insulin, leptin, Pyy and Acth levels are increased in mutant mice (D,E,G,H), whereas adiponectin levels are unaffected (F). *P<0.05, **P<0.01, ***P<0.005.

Owing to the role of *Ngn3* in glucose homeostasis, and the association of obesity with endocrine disorders such as type 2 diabetes, we next investigated in *Nkx2.1iCre/+;Ngn3^flox/flox^ mice* the glucose/insulin response system as well as a number of other hormones associated with obesity (in the same cohort of *n*=9 for each sex and genotype). Following glucose and insulin challenge, mutant mice were significantly more intolerant of glucose compared with controls (Fig. 4A,B), with the effect being more pronounced in females than in males. Insulin challenge revealed that female mutant mice were significantly more insensitive to insulin compared with controls, whereas male mice show elevated insulin tolerance but not as to reach statistical significance (Fig. 4C). Furthermore, blood analysis demonstrated that male mutant mice had significantly elevated blood insulin compared with controls, with female mice showing a large difference approaching significance (Fig. 4D). Blood leptin was significantly increased in both male and female mutant animals compared with controls (Fig. 4E), due to increased body fat. Conversely, no change in adiponectin was detected in either sex (Fig. 4F). Compared with controls, peptide YY (Pyy) levels were increased in both males and females, but not as to reach statistical significance (Fig. 4G). This is in part due to a high standard deviation in mutant sample, particularly in male mice. Levels of adrenocorticotropic hormone (Acth), a Pomc product released by the pituitary, were significantly higher in mutant female mice than in controls, and were elevated, but not statistically significantly so, in males (Fig. 4H). This data suggests that *Pomc* expression, and hence Acth production, is deregulated in the pituitary as a consequence of the loss of *Ngn3* expression in Acth-secreting pituitary cells. However, nuclear expression of *Ngn3* is not detectable by immunohistochemistry in the pituitary, and qRT-PCR expression suggests that, when present, *Ngn3* is expressed at only very low levels (Fratticci et al., 2007). It is therefore unlikely that loss of Ngn3 in the pituitary is responsible for the elevation of serum Acth, and so an indirect cause must be sought.

### Arcuate *Nhlh2* expression is absent in *Nkx2.1iCre/+;Ngn3^flox/flox^* mice

**Fig. 5.**
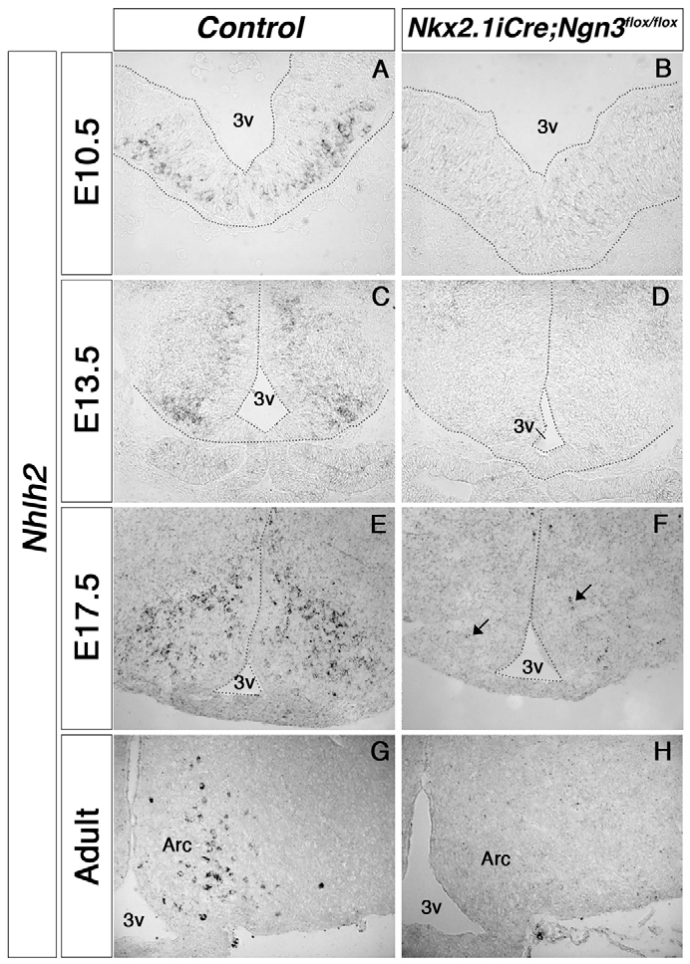
**Expression of *Nhlh2* in the hypothalamus of developing and adult *Nkx2.1iCre/+;Ngn3^flox/flox^* mice. ***In situ* hybridization reveals that *Nhlh2* expression is absent in the early embryonic hypothalamus of mutant mice (A–D). In late development, a few *Nhlh2*-positive cells are observed per section (E,F; arrows); however, no expression is observed in the adult mutant ARC (G,H). 3v, third ventricle; Arc, arcuate nucleus.

Nhlh2 is a bHLH transcription factor expressed in the developing and adult hypothalamus, including in the ARC. *Nhlh2*-null mice become obese at postnatal week 8 (Coyle et al., 2002), and we have previously demonstrated reduced expression of *Nhlh2* in *Ngn3*-null mice (Pelling et al., 2011). *Nhlh2* expression was severely reduced in *Nkx2.1iCre/+;Ngn3^flox/flox^* mutants. We determined the expression of *Nhlh2* by *in situ* hybridization in hypothalami of embryonic and adult *Nkx2.1iCre/+;Ngn3^flox/flox^* mutants. Deletion of *Ngn3* in the ventral forebrain resulted in loss of *Nhlh2* expression in the presumptive hypothalamus at E10.5 and E13.5 (Fig. 5A–D). Although sparse *Nhlh2* expression was transiently observed in E17.5 embryos (Fig. 5E,F), expression of this gene was absent in adult mutants (Fig. 5G,H). These results confirm earlier studies (Pelling et al., 2011) and further indicate that *Nhlh2* expression is lost in the adult hypothalamus in the absence of Ngn3 activity during embryonic stages.

### Deletion of *Ngn3* results in a reduction in the number and incomplete specification of *Pomc/Cart* neurons

**Fig. 6.**
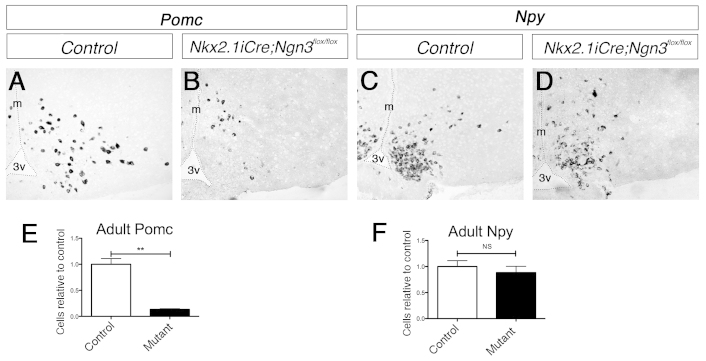
**Expression by *in situ* hybridization of *Pomc* and *Npy* in the hypothalamus of *Nkx2.1iCre/+;Ngn3^flox/flox^* and control mice.** (A–D) Anorexigenic *Pomc* expression compared with the expression in control mice is reduced in the ARC (A,B), whereas orexigenic *Npy* expression is unchanged (C,D). (E,F) Quantification of the number of *Pomc*- and *Npy*-expressing neurons in the ARC of *Nkx2.1iCre/+;Ngn3^flox/flox^* mice compared with control mice. The number of *Pomc*^+^ cells is reduced by around 80% in *Nkx2.1iCre/+;Ngn3^flox/flox^* mutant mice (E), whereas the number of *Npy*^+^ cells is unchanged (F). Student’s *t*-test: NS, not significant; **P<0.01.

We have previously demonstrated that *Ngn3*-null mutant embryos show a substantial reduction of postmitotic *Pomc^+^* cells from the initial expression at E10.5, with the perinatal (E17.5) number of *Pomc^+^* neurons being 60–70% of that seen in wild types (Pelling et al., 2011). In the present study, *Nkx2.1iCre/+;Ngn3^flox/flox^* embryos showed a similar reduction of hypothalamic *Pomc*^+^ postmitotic neurons during embryonic development (supplementary material Fig. S1). The reduction in *Pomc^+^* cells remained into adulthood in *Nkx2.1iCre/+;Ngn3^flox/flox^* mice, with the total number of neurons being around 20% of that seen in control hypothalami (Fig. 6A,B,E). Compared with controls, no substantial difference was observed in the number of *Pomc*^+^ cells in the ARC of male or female mutant mice. Importantly, expression of the Pomc product α-melanocyte stimulating hormone (αMsh) was almost completely abolished in mutants, with only a few expressing cells being observed throughout the ARC (supplementary material Fig. S2). *Ngn3*-null mice also displayed an accompanying increase in *Npy*^+^ cells in the ARC that is suggestive of a change in fate of developing ARC cells from *Pomc*-expressing to *Npy*-expressing (Pelling et al., 2011). However, *Nkx2.1iCre/+;Ngn3^flox/flox^* mutant embryos or adult mice did not exhibit any difference in the number of *Npy*-expressing cells compared with controls, although the pattern of *Npy* expression was less compacted in *Nkx2.1iCre/+;Ngn3^flox/flox^* adult mice compared with control mice (Fig. 6C,D,F; supplementary material Fig. S1). Furthermore, despite the reduction in *Pomc*^+^ cells and the normal number of *Npy*^+^ neurons in the adult hypothalamus, there was no obvious change in the cytoarchitecture of the hypothalamus (data not shown).

**Fig. 7.**
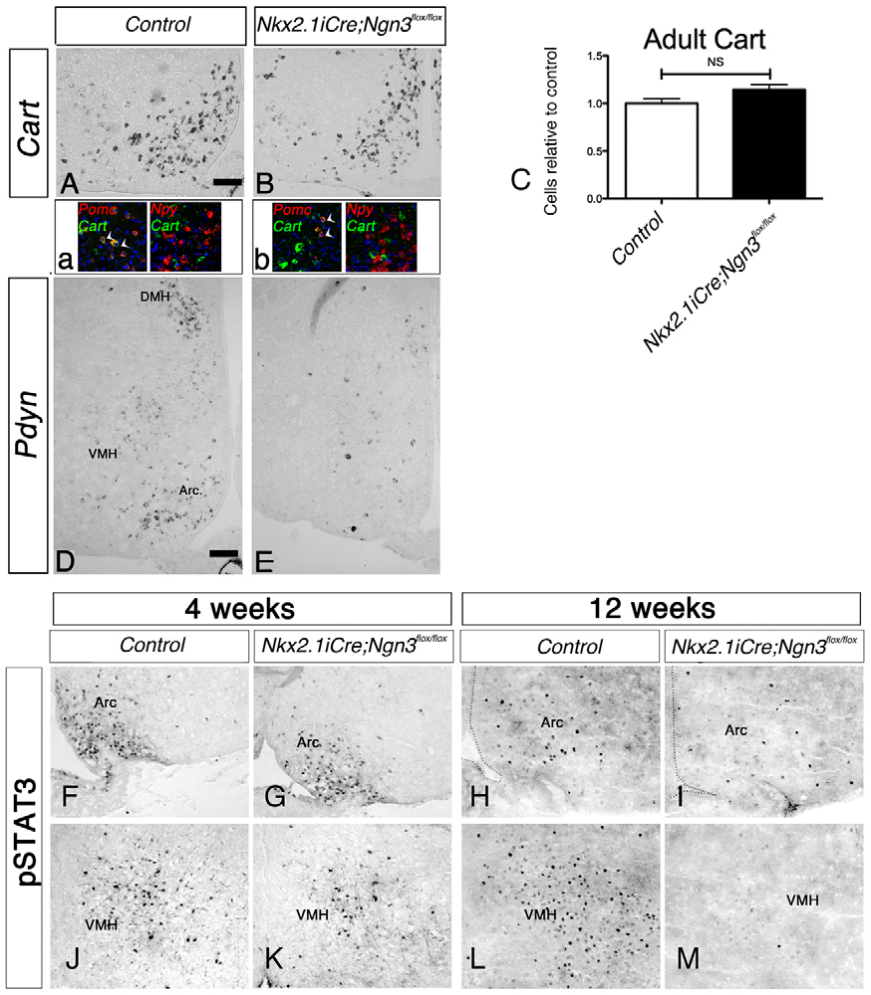
**Analysis of *Cart* and *Pdyn* expression by *in situ* hybridization, and of pSTAT3 by immunohistochemistry, in *Nkx2.1iCre/+;Ngn3^flox/flox^* and control mice.** (A–E) Hypothalamic expression analyzed by *in situ* hybridization of *Cart* and *Pdyn* in adult *Nkx2.1iCre/+;Ngn3^flox/flox^* mice. (A,B) *Cart* expression is largely unchanged in mutant mice compared with controls. Double-fluorescence *in situ* hybridization (a,b) shows that *Cart* is always coexpressed with *Pomc* in the ARC of control mice, whereas both *Cart/Pomc* double-labeled cells (arrowheads) and *Cart* single-labeled cells are observed in the mutant condition. *Cart* is never coexpressed with *Npy* in control or mutant mice. (C) Quantification by cell counting shows that the number of *Cart*^+^ cells in adult mutant mice is unaffected. (D,E) Expression of *Pdyn*, the gene encoding the opioid dynorphin, is reduced in the VMH, DMH and ARC, where it is coexpressed by a subset of POMC^+^ neurons, in the conditional mutant mice compared with controls. (F–M) Immunohistochemical analysis of the expression of pSTAT3 following leptin challenge in 4-week- and 12-week-old *Nkx2.1iCre/+;Ngn3^flox/flox^* mutant mice. pSTAT3 is a intracellular effector of LepR. (F,G,J,K) Expression is unaffected in the ARC (F,G) and VMH (J,K) of pre-obese mutant mice at 4 weeks of age. (H,I,L,M) Obese 12-week-old mutant mice display reduced pSTAT3 expression in both the ARC (H,I) and VMH (L,M), demonstrating the onset of leptin insensitivity. DMH, dorsomedial hypothalamic nucleus; VMH, ventromedial hypothalamic nucleus; Arc, arcuate nucleus.

We next determined whether the loss of Pomc expression was due to gene regulation or to a loss of neurons. *Cart* transcripts were found in the *Pomc*-expressing neurons of the ARC of adult control mice. In *Nkx2.1iCre/+;Ngn3^flox/flox^* mice, *Cart* expression was maintained in the ARC (Fig. 7A–C). To further probe the identity of the *Cart*^+^ cells, double-fluorescent *in situ* hybridization for *Cart* and *Pomc*, and for *Cart* and *Npy*, was carried out. Many *Cart*^+^ cells in the ARC of *Nkx2.1iCre/+;Ngn3^flox/flox^* mice did not coexpress *Pomc*, whereas all arcuate *Cart*^+^ neurons coexpressed *Pomc* in control mice (Fig. 7A,B). Furthermore, as in the control mice, there was no overlap of *Cart* and *Npy* transcript expression in the mutant ARC (Fig. 7A,B)

A proportion of *Pomc*^+^ arcuate neurons also express the endogenous opioid dynorphin (Maolood and Meister, 2008), which itself is implicated in energy balance and whose expression is sensitive to leptin levels (Levin and Dunn-Meynell, 2002; Silva et al., 2002). *In situ* hybridization for *Pdyn*, the gene encoding the dynorphin protein, was carried out in adult control and conditional mutant hypothalamic sections (Fig. 7D,E). Expression of *Pdyn* was substantially reduced throughout the hypothalamus, not only in the ARC but also in the VMH and the DMH hypothalamus. Because the DMH is not derived from Ngn3^+^ progenitors (data not shown), it is likely that the reduction in *Pdyn* expression is secondary to elevated blood leptin. The ARC also includes a population of tyrosine hydroxylase (TH)-expressing dopaminergic neuroendocrine cells, the number of which is increased in *Ngn3*-null mutants (Pelling et al., 2011). However, *in situ* hybridization for *Th* mRNA revealed that there was no obvious change in the number of dopaminergic neurons in the ARC of *Nkx2.1iCre/+;Ngn3^flox/flox^* mutants (see supplementary material Fig. S2A,B). The ARC is also an important part of the hypothalamic-pituitary-gonadal axis, and includes a population of estrogen receptor-α (ERα)-expressing neurons, a subset of which colocalize with *Pomc*^+^ neurons (de Souza et al., 2011). To determine whether the expression of ERα is affected in *Nkx2.1iCre/+;Ngn3^flox/flox^* mice, fluorescent immunohistochemistry was carried out (see supplementary material Fig. S2C,D). No difference was observed in the pattern or intensity of staining at the level of the ARC between control and mutant mice. These expression patterns (*Th*, *Pdyn*, *Cart* and ERα) were similar in both male and female mice of both genotypes.

In summary, neurons expressing *Pomc* were reduced by 80% in the ARC of *Nkx2.1iCre/+;Ngn3^flox/flox^* mice, and the Pomc subset marker *Pdyn* was severely reduced, whereas *Cart* expression was not affected. Expression of other arcuate markers – *Npy*, *Th* and ERα – were unchanged.

### Leptin response is altered in the VMH and ARC

Leptin is a hormone that is released by fat tissue and is known to have an important regulatory effect on the central control of feeding (Elmquist et al., 1999; Schwartz et al., 2000). LepRs are expressed in the brain, including in the VMH and ARC (Scott et al., 2009). Binding of leptin to the LepR leads to activation of the intracellular JAK-STAT pathway via the phosphorylation of the STAT3 transcription factor (Ghilardi and Skoda, 1997). In order to detect any changes in leptin response in the hypothalamus in the absence of *Ngn3*, pSTAT3 expression was analyzed in leptin-treated control and *Nkx2.1iCre/+;Ngn3^flox/flox^* mice. To avoid any secondary leptin insensitivity caused by obesity, 4-week-old pre-obese mice in addition to 12-week-old obese animals were examined (Fig. 7F–M). In 4-week-old mice, pSTAT3^+^ cells were similarly observed in the VMH and ARC region of both control and mutant mice (Fig. 7F,G,J,K). However, a slight reduction in pSTAT3 signal was observed in the ARC of obese conditional mutants at 12 weeks of age (Fig. 7H,I), in line with the previously described situation in diet-induced obese (DIO) models (Münzberg et al., 2004; Mitchell et al., 2009). Interestingly, expression in the VMH was greatly reduced in obese conditional mutants (Fig. 7L,M), contrary to the DIO models in which VMH is spared from leptin insensitivity. These results suggest that the leptin insensitivity is a consequence rather than the cause for obesity in *Nkx2.1iCre/+;Ngn3^flox/flox^* animals.

## DISCUSSION

### Arcuate anorexigenic neurons are present but not completely differentiated in adult *Nkx2.1iCre/+;Ngn3^flox/flox^* mice

Conditional deletion of *Ngn3* results in an 80% reduction in *Pomc* expression in the ARC. Interestingly, *Cart*^+^ neurons persist in the brains of adult *Nkx2.1iCre/+;Ngn3^flox/flox^* mice. This includes a majority of *Cart*^+^*Pomc*^−^ neurons as well as a small number of *Cart*^+^*Pomc^+^* in the *Nkx2.1iCre/+;Ngn3^flox/flox^* ARC, whereas all ARC *Cart*^+^ cells coexpress *Pomc* in control mice, consistent with previous expression studies (Vrang et al., 1999). This suggests that ARC anorexigenic neurons are generated in *Nkx2.1iCre/+;Ngn3^flox/flox^* mutants, albeit that a subset of these neurons do not express *Pomc* and *Pdyn*. Altogether, these results strongly suggest that *Ngn3* contributes to the specification of ARC anorexigenic neurons.

The ARC of *Ngn3*-null mice (Pelling et al., 2011) seems to have a compensatory increase in *Npy*-expressing neurons, which is not observed in *Nkx2.1iCre/+;Ngn3^flox/flox^* mice. This partial phenocopy of the *Ngn3*-null mouse might be explained by the incomplete deletion of *Ngn3* at E9.5 (Fig. 1). We therefore suggest that a low level of *Ngn3* expression is able to drive putative ARC precursors towards an anorexigenic fate rather than an *Npy*^+^ orexigenic fate. However, the lack of sustained *Ngn3* expression results in these anorexigenic neurons failing to fully differentiate and express Pomc. Interestingly, the number of *Pomc*^+^ neurons partially recovers during development of the *Ngn3*-null mouse (Pelling et al., 2011), and yet no such recovery is seen in the hypothalamus-specific mutants. The cause for these phenotypic differences is currently unknown and warrants further study.

### *Nkx2.1iCre/+;Ngn3^flox/flox^* obesity is primarily due to loss of Pomc products specifically in the ARC

The absence of hypothalamic *Ngn3* during development results in a partial specification of ARC neurons with the consequence that *Pomc*^+^ neurons are largely absent. The resulting obesity can be attributed primarily to the loss of Pomc active peptides. *Pomc*-null mutant mice are hyperphagic and obese (Yaswen et al., 1999), although not to the extent seen in *Nkx2.1iCre/+;Ngn3^flox/flox^* mice. *Pomc*-null mice also exhibit no elevation in basal insulin levels, have normal glucose tolerance and show increased insulin sensitivity (Hochgeschwender et al., 2003). However, this surprising non-diabetic phenotype is explained by the loss of the Pomc product peptide Acth in the pituitary and subsequent hypocortisolism of *Pomc*-null mice. When *Pomc* expression is rescued by transgenic insertion of the *Pomc* gene specifically in the pituitary of *Pomc*-null mutants, the resulting *Pomc^−/−^Tg^+^* mice show amelioration of the deficiencies due to loss of peripheral Pomc, such as adrenal gland failure and altered glucose homeostasis (Smart et al., 2006). When compared with *Pomc^−/−^* mice, obesity in *Pomc^−/−^Tg^+^* mutants is increased and the rate of weight gain is more rapid. There is an increase in food intake in *Pomc^−/−^Tg^+^* compared with global *Pomc* nulls, the metabolic rate is decreased as indicated by reduced oxygen consumption (VO_2_) and an increased respiratory exchange ratio (referred to as RQ in this instance), and basal insulin levels are increased in obesity (Smart et al., 2006). More recently, arcuate-specific *Pomc* mutant mice have been generated, which have rapid post-weaning obesity and associated hyperglycemia and elevated leptin (Bumaschny et al., 2012). These phenotypes of rapid post-weaning obesity associated with hyperphagia and decreased metabolism are remarkably similar to those seen in the *Nkx2.1iCre/+;Ngn3^flox/flox^* mice of the current study, strongly suggesting that the primary cause of obesity in *Nkx2.1iCre/+;Ngn3^flox/flox^* mice is due to loss of hypothalamic Pomc.

### Additional axes of energy balance affected in *Ngn3* conditional mutant mice

The current study presents a model for post-weaning obesity associated with hyperphagia and correlated with reduced energy expenditure. Yet we have only demonstrated a reduction in energy expenditure (via locomotor activity and indirect calorimetry) in animals that are already obese, allowing for the possibility that the reduction observed in energy expenditure is secondary to hyperphagic obesity. However, although it might be true that the early increase in weight observed is due to excess feeding, genetic evidence suggests that reduced energy expenditure might be a primary phenotype associated with the loss of *Nhlh2* expression (Coyle et al., 2002). In addition, our results strongly suggest that Ngn3 has an additional role in regulating energy expenditure through Nhlh2, whose expression is severely reduced in ARC neurons throughout embryonic development and in adult *Nkx2.1iCre/+;Ngn3^flox/flox^* mice. Furthermore, there might also be a compounded reduction in energy expenditure resulting from the later insensitivity of the VMH to leptin. The mechanism underlying this leptin insensitivity is currently unknown.

There is also a trend in *Nkx2.1iCre/+;Ngn3^flox/flox^* mutants for an increase in circulating Pyy. Pyy is released by *Ngn3*-dependent intestinal endocrine glands following feeding and acts to inhibit feeding by binding Npy receptors. It is likely that the elevation of Pyy is part of the normal homeostatic response trying to reduce appetite (Karra et al., 2009). Likewise, the levels of circulating leptin and insulin are significantly increased. Unlike the unexpected elevated circulating Acth levels, these responses are part of the normal homeostatic response to excess obesity.

### Ngn3 is a regulator of energy homeostasis systems throughout the body, and is a candidate for genetic obesity and diabetes in humans

The importance of *Ngn3* in energy homeostasis is well established via its role in pancreas islet cell and gut endocrine cell specification (Gradwohl et al., 2000; Schonhoff et al., 2004; Mellitzer et al., 2010). The current study, as well as the embryonic analysis of the *Ngn3*-null mouse embryos (Pelling et al., 2011), highlights the importance of *Ngn3* in the development of the brain component of the feeding control circuit. It is noteworthy that the same transcription factor is involved in the specification of tissues involved in energy homeostasis in both the brain and the endocrine system, although *Ngn3* acts as a proendocrine specifier in the pancreas and intestine, whereas in the hypothalamus it gives rise to multiple neuronal fates, not only neuroendocrine. As yet, no firm link between *Ngn3* and type 2 diabetes has been described in humans; however, the link between obesity and type 2 diabetes is well known. Obesity and associated diseases such as type 2 diabetes are now at epidemic levels in western societies, due in part to increased energy consumption and sedentary lifestyle. However, there is still variation in obesity rates within populations living similar lifestyles. There must therefore be a genetic or inherent component to the obesity epidemic. The current understanding of the basis of genetic obesity is patchy, although a number of single-nucleotide polymorphisms (SNPs) are known. Although *Ngn3* disruption in the endocrine system seems to have a greater effect upon patient outcomes for those few known cases than any changes in the hypothalamus (Wang et al., 2006; Pinney et al., 2011), the direct targets of *Ngn3* in the hypothalamus are still unclear. As such, better understanding of the action of *Ngn3* within the hypothalamus might illuminate further the basis of genetic obesity in humans.

## MATERIALS AND METHODS

### Generation of conditional knockout mice

In order to study the consequence of deletion of *Ngn3* in the hypothalamus throughout development, mice that are homozygous for the floxed *Ngn3* allele (*Ngn3^flox/flox^*) were generated as previously described (Mellitzer et al., 2010). Female *Ngn3^flox/flox^* mice were then crossed with male mice carrying a single copy of the *Nkx2.1iCre* allele. The resulting male mice were crossed again with *Ngn3^flox/flox^* female mice to generate homozygous conditional-knockout *Nkx2.1iCre/+;Ngn3^flox/flox^* mutant mice, and control *Ngn3^flox/flox^* littermates.

### qRT-PCR

Control and mutant embryos were collected at E9.5 (*n*=3), and the ventral forebrain was immediately dissected and collected into 0.5 ml tubes containing 50 μl of Arcturus PicoPure extraction buffer, and RNA extracted using the Arcturus PicoPure RNA isolation system (Applied Biosystems, Carlsbad, CA). Similarly, E15.5 pancreata (*n*≥3) were dissected and immediately placed in 100 μl of extraction buffer and RNA was extracted in the same manner. cDNA was made using Superscript III First Strand Synthesis kit (Life Technologies, Paisley, UK). qRT-PCR was carried out using the following primer pair against *Ngn3* mRNA: Fwd 5′-GGATGACGCCAAACTTACAAA-3′, Rev 5′-AGAAGCTGTGGTCCGCTATG-3′. *Ngn3* levels were then expressed as % *Gapdh* [100×2^−ΔCT)^] by normalization against *Gapdh* expression detected with the following primer pair: Fwd 5′-AATCCCATCACCATCTTCCA-3′, Rev 5′-GGCAGTGATGGCATGGACTG-3′.

### Slide preparation

Embryonic tissue was prepared for *in situ* hybridization and immunohistochemistry in the following manner. Whole embryos were dissected at E9.5, E10.5 and E13.5, and embryonic brains dissected at E15.5 and E17.5, and fixed overnight in 4% paraformaldehyde (PFA), before being washed in DEPC-treated PBS and cryopreserved overnight in filtered 30% sucrose/DEPC-treated PBS. Tissue was then embedded in OCT medium, frozen for coronal sectioning on dry ice and stored at −20°C or −80°C until required. Adult mice were placed into terminal anesthesia by an intraperitoneal (i.p.) injection of 50 mg pentobarbital/kg body weight. Once fully anesthetized, mice were transcardially perfused with 0.5% saline followed by 25–50 ml of 4% PFA depending on body mass. Once perfusion was complete, the brains and large intestines were collected and post-fixed overnight in 4% PFA, before being processed for cryosectioning as described above. Coronal sections were made at −20°C with a cryostat (Leica, Nussloch, Germany) and hypothalamic tissue mounted on Superfrost Plus slides, air dried and stored at −80°C until required. E9.5 and E10.5 embryos were sectioned at 10 μm and mounted over a series of four slides; E13.5 and E15.5 embryos were sectioned at 12 μm and mounted over a series of six slides; and E17.5 and postnatal stages were sectioned at 14 μm and mounted over a series of eight slides. In order to minimize variations in gene expression due to normal diurnal changes, all tissue was collected between 1 and 3 hours before the onset of the normal 12-hour dark phase (2–4 pm).

### *In situ* hybridization and immunohistochemistry

*In situ* hybridization was carried out as previously described (Vernay et al., 2005). The following antisense RNA probes were used: *Ngn3* (Gradwohl et al., 2000), *Pomc* (Good et al., 1997), *Npy* (Higuchi et al., 1988), *Nhlh2* (Good et al., 1997), *Cart* (Image clone #466874), *Th* (Grima et al., 1985). An antisense mRNA probe against *Pdyn* was made from a cDNA template as previously described (Krawchuk and Kania, 2008). The cDNA was derived from adult mouse hypothalamus tissue and the probe generated using the following primer pair: Fwd 5′-CCCCCTGATTTGCTCCCTGGAGT-3′, Rev (including T7 site) 5′-GGTAATACGACTCACTATAGGGTGAACTGACGCCGCAGGAAACC-3′. Two-color fluorescence *in situ* hybridization was carried out with digoxygenin-labeled antisense mRNA probes against *Cart* and fluorescein-labeled probes against either *Pomc* or *Npy* and revealed with fast red and Alexa-Fluor-488-conjugated TSA kit (Life Technologies) as previously described (Ishii et al., 2004), before mounting with Prolong Gold antifade reagent with DAPI (Life Technologies).

For immunohistochemistry to detect Erα and αMSH, sections were incubated overnight at 4°C with rabbit anti-Erα primary antibody (Millipore, Temecula, CA) diluted 1/200 in blocking solution (0.1% Triton X-100 and 1% BSA in PBS) or rabbit anti-αMSH primary antibody (Millipore) diluted 1/10,000 in blocking solution. Sections were then extensively washed in PBS and incubated for 2 hours at room temperature with a donkey anti-rabbit secondary antibody conjugated with Fitc (for Erα) or Cy3 (for αMSH) (both Stratech, Newmarket, UK). Sections were then extensively washed and mounted in Prolong Gold antifade reagent with DAPI (Life Technologies, Paisley, UK). Immunohistochemistry using anti-pSTAT3 (Cell Signaling, Danvers, MA) was carried out as previously described (Münzberg et al., 2003) with the following modifications: overnight fasted mice were treated by i.p. injection with 5 mg human recombinant leptin/kg body weight (R&D Systems, Abingdon, UK) 45 minutes before being perfused with 2% PFA, post-fixed overnight and processed to generate slides. Defrosted sections were then washed in PBS followed by a 30-minute treatment in 50% ice-cold methanol, 5% H_2_O_2_. Slides were then washed extensively and incubated overnight at 4°C with rabbit anti-pSTAT3 primary antibody diluted in blocking solution. Washed sections were then incubated for 2 hours with biotinylated anti-rabbit secondary antibodies (Vector Laboratories, Peterborough, UK), the signal amplified with ABC Vecastain kit (Vector Laboratories) and revealed with DAB-Ni (Vector Laboratories). Slides were then dehydrated through a graded ethanol series to prevent loss of DAB-Ni stain, and mounted in DePX mounting medium.

All immunohistochemistry and *in situ* hybridization experiments were repeated with tissue from at least three separate individuals (*n*≥3), and in both male and female mice.

### Image analysis and cell counting

Fluorescence *in situ* hybridization and immunohistochemistry images were then obtained with a Leica TCS SP2 (Leica) confocal microscope. Colorimetric stains were imaged with a Zeiss LSM510 microscope (Carl Zeiss, Oberkochen, Germany) connected to a computer running Axioplan2.1 imaging software. Images were processed with Adobe Photoshop CS5 and NIH ImageJ (http://rsbweb.nih.gov/ij/).

All cell counts were carried out using the Cell Counter plug-in for ImageJ. Cell counts were derived from an average of three counts of whole cell nuclei from a single series of cryostat-cut sections per animal, with a mean taken from a minimum of three series per expression pattern of interest (*n*≥3). All positive cells from either the left or right side hemisphere were counted across the whole anterior-posterior axis of the ARC each series. Only ARC *Npy*-expressing cells were counted, excluding those found in the DMH and elsewhere. All counts were carried out with the operator blind as to the genotype of the sample being counted. Student’s *t*-test was then carried out to compare the control and conditional mutant counts for statistical significance (*P*<0.05).

### Physiological and behavioral assays

All physiological and behavioral studies were carried out on the same cohort of 36 mice transported at 8 weeks of age from the UK to the Mouse Clinical Institute, Illkirch, France. This cohort included 18 *Nkx2.1iCre/+;Ngn3^flox/flox^* mice and 18 control (*Ngn3^flox/flox^*) controls; 9 males and 9 females of each genotype. Body weight was measured weekly from the age of 12 to 22 weeks, and food intake was measured daily between the age of 12 to 14 weeks, with the mice housed one to three individuals per cage. For paired-feeding experiments, daily food consumption of individually housed *ad libitum*-fed control mice was measured (*n*=3) between 4 and 6 weeks of age. Individually housed *Nkx2.1iCre/+;Ngn3^flox/flox^* littermate mice were then either restricted in their food by the amount consumed by the control mice the previous night (*n*=3), or allowed to feed *ad libitum* (*n*=3). All mice were weighed at the beginning of the experiment followed by twice-weekly measurements and the mean weight gain compared. Mice were fed standard lab chow throughout the experiment (Labdiet, Autoclavable Laboratory Animal Diet 5021).

### Body composition

Body composition was measured in conscious 14-week-old mice (*n*=9 for each sex and genotype) by qNMR using the Minispec analyzer (Bruker, Karlsruher, Germany).

### Energy expenditure

Following body composition analysis, energy expenditure was then evaluated at 17 weeks by O_2_ consumption, CO_2_ production, heat production and activity, monitored using the TSE Labmaster (TSE Systems, Bad Homburg, Germany) (*n*=9 for each sex and genotype). Following 24 hours acclimatization, analysis was carried out over a 24-hour period including a 12-hour dark photoperiod and 12 hours light.

### Intraperitoneal glucose tolerance test and insulin sensitivity test

Glucose and insulin sensitivity was measured in the same cohort of mice (*n*=9 for each sex and genotype) at 19 and 21 weeks by i.p. injection of a standardized 2 g dose of glucose/kg body weight (at 19 weeks) or 0.5 UI insulin/kg body weight (at 21 weeks) following a period of fasting (16 hours glucose tolerance test, 2 hours insulin sensitivity test). Following i.p. challenge, blood was obtained from the tail and glucose was measured by use of a blood glucose monitor and glucose test strips (Roche Diagnostics, Accu-Chek) at different time points (over 180 minutes in the case of the glucose tolerance test, and 90 minutes in the insulin sensitivity test). All tests were conducted during the light period.

### Blood analysis

Blood was collected from 23-week-old mice (*n*=9 for each sex and genotype) by retro orbital puncture under isoflurane anesthesia following 4 hours of fasting. Plasma insulin, leptin and Pyy were measured with a BioPlex analyzer using Milliplex beads (Millipore). Adiponectin was measured by ELISA using the Quantikine Adiponectin/Acrp30 immunoassay (R&D Systems). Acth was measured by RIA using the ImmunoChem double antibody Acth ^125^I RIA kit (MP Biomedical).

### Statistical analysis

All comparisons between *Nkx2.1iCre/+;Ngn3^flox/flox^* mice and controls were performed using ANOVA test followed by Student’s *t*-test.

## Supplementary Material

Supplementary Material
