# Supplementary material for: Conditional deletion of neurogenin-3 using Nkx2.1iCre results in a mouse model for the central control of feeding, activity and obesity
Source: Dis Model Mech. 2013 May 2;6(5):1133–45. doi: 10.1242/dmm.011916 (PMC3759333; doi:10.1242/dmm.011916)
Supplement: Supplementary Material [file supp_6_5_1133__index.html]

Conditional deletion of neurogenin-3 using Nkx2.1iCre results in a mouse model for the central control of feeding, activity and obesity — Conditional deletion of neurogenin-3 using Nkx2.1iCre results in a mouse model for the central control of feeding, activity and obesity — Supplementary Material 

# Conditional deletion of neurogenin-3 using *Nkx2.1iCre* results in a mouse model for the central control of feeding, activity and obesity

## 

**Files in this Data Supplement:**

- **Supplementary Material PDF**
