# Supplementary material for: Conditional deletion of neurogenin-3 using Nkx2.1iCre results in a mouse model for the central control of feeding, activity and obesity
Source: Dis Model Mech. 2013 May 2;6(5):1133–45. doi: 10.1242/dmm.011916 (PMC3759333; doi:10.1242/dmm.011916)
Supplement: Supplementary Material [file supp_011916_DMM011916.pdf]

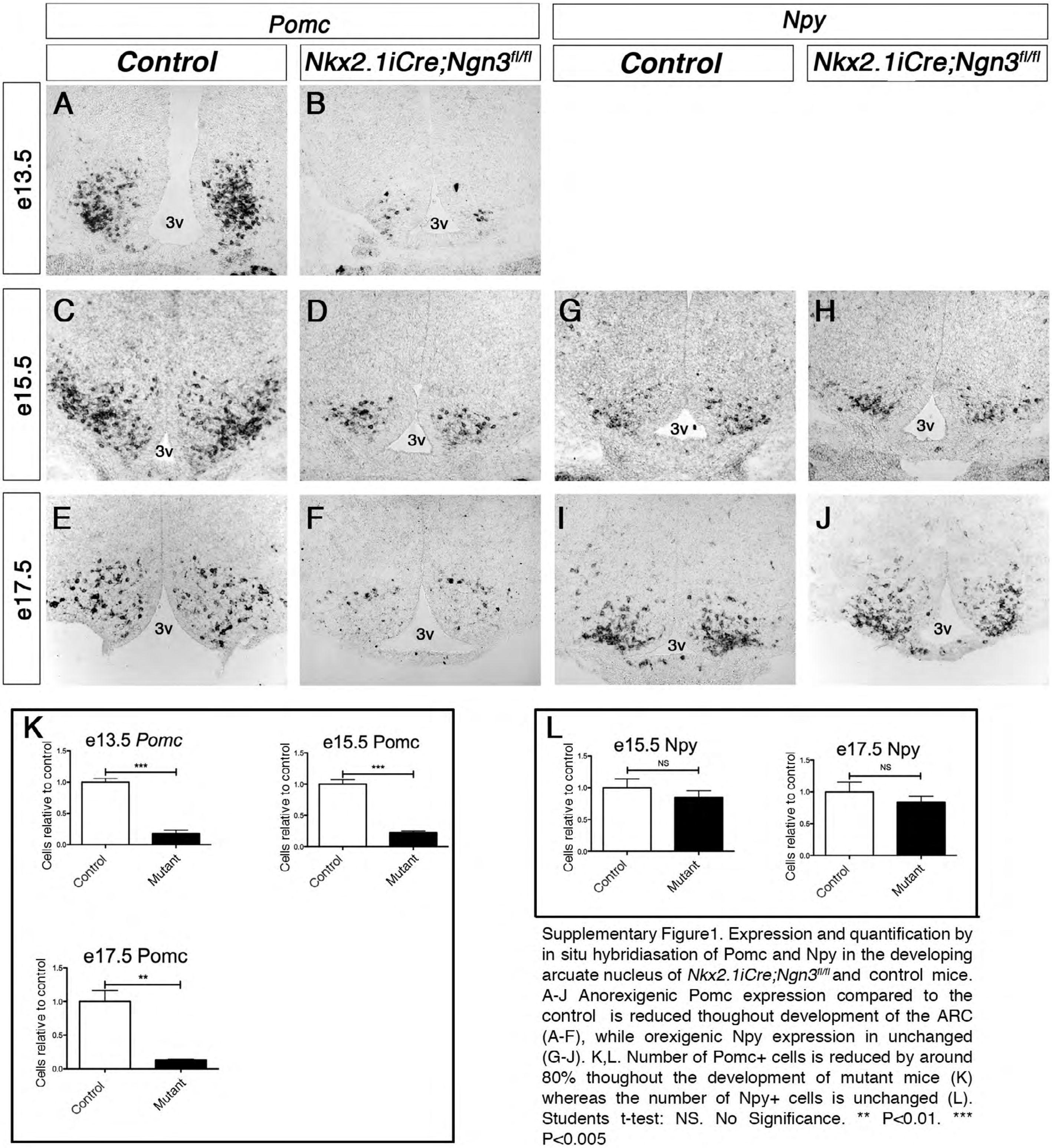

Supplementary Figure1. Expression and quantification by in situ hybridiasation of Pomc and Npy in the developing arcuate nucleus of *Nkx2.1iCre;Ngn3$^{fl/fl}$* and control mice. A-J Anorexigenic Pomc expression compared to the control is reduced thoughout development of the ARC (A-F), while orexigenic Npy expression in unchanged (G-J). K,L. Number of Pomc+ cells is reduced by around 80% thoughout the development of mutant mice (K) whereas the number of Npy+ cells is unchanged (L). Students t-test: NS. No Significance. ** P<0.01. *** P<0.005

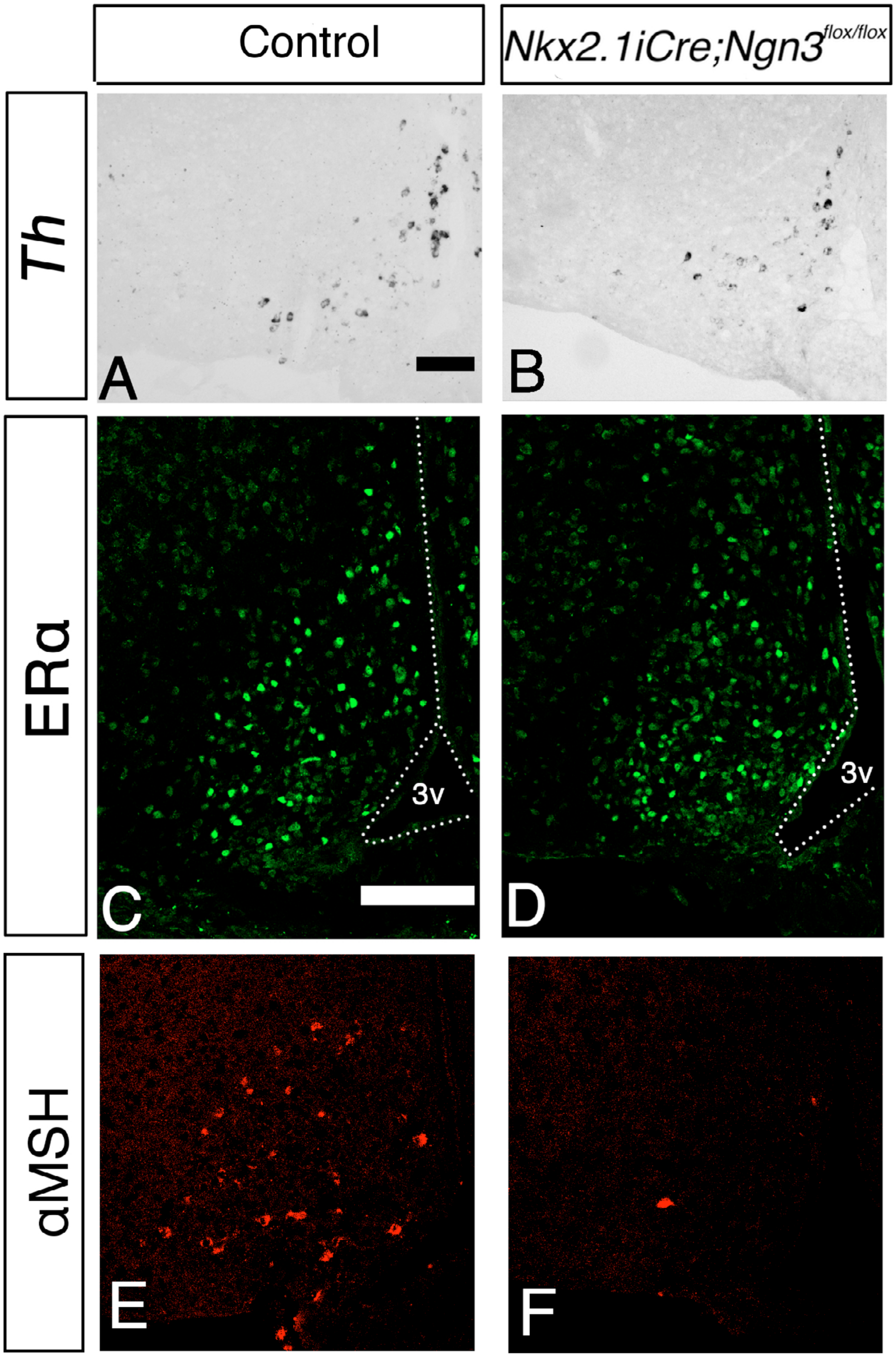

Supplementary Figure 2.
Expression by in situ hybridisation of *Th* and by flourescence immunohistochemistry of ERα and αMSH in the arcuate nucleus of control and *Nkx2.1iCre;Ngn3*$^{flox/flox}$ mice. Expression of *Th* (A,B) and of ERα (C,D) is unaffected in mutant mice, while αMSH expression is largely absent. 3v: 3rd ventricle.
